# In marine *Bacteroidetes* the bulk of glycan degradation during algae blooms is mediated by few clades using a restricted set of genes

**DOI:** 10.1038/s41396-019-0476-y

**Published:** 2019-07-17

**Authors:** Karen Krüger, Meghan Chafee, T. Ben Francis, Tijana Glavina del Rio, Dörte Becher, Thomas Schweder, Rudolf I. Amann, Hanno Teeling

**Affiliations:** 1 Max Planck Institute for Marine Microbiology, Celsiusstraße 1, 28359 Bremen, Germany; 2 DOE Joint Genome Institute, 2800 Mitchell Drive, Walnut Creek, CA 94598 USA; 3 Institute for Microbiology, University Greifswald, Felix-Hausdorff-Straße 8, 17489 Greifswald, Germany; 4 Pharmaceutical Biotechnology, Institute of Pharmacy, University Greifswald, Felix-Hausdorff-Straße 3, 17487 Greifswald, Germany; 5 Institute of Marine Biotechnology, Walther-Rathenau-Straße 49a, 17489 Greifswald, Germany

**Keywords:** Microbial ecology, Proteomics, Biodiversity, Metagenomics

## Abstract

We investigated *Bacteroidetes* during spring algae blooms in the southern North Sea in 2010–2012 using a time series of 38 deeply sequenced metagenomes. Initial partitioning yielded 6455 bins, from which we extracted 3101 metagenome-assembled genomes (MAGs) including 1286 *Bacteroidetes* MAGs covering ~120 mostly uncultivated species. We identified 13 dominant, recurrent *Bacteroidetes* clades carrying a restricted set of conserved polysaccharide utilization loci (PULs) that likely mediate the bulk of bacteroidetal algal polysaccharide degradation. The majority of PULs were predicted to target the diatom storage polysaccharide laminarin, alpha-glucans, alpha-mannose-rich substrates, and sulfated xylans. Metaproteomics at 14 selected points in time revealed expression of SusC-like proteins from PULs targeting all of these substrates. Analyses of abundant key players and their PUL repertoires over time furthermore suggested that fewer and simpler polysaccharides dominated early bloom stages, and that more complex polysaccharides became available as blooms progressed.

## Introduction

Spring and summer blooms of planktonic microalgae (phytoplankton) are annually recurring phenomena in the world’s oceans’ temperate coastal regions. Such blooms can vary in overall phytoplankton composition, and likewise the composition within an individual bloom can undergo multiple successive changes over its course. These dynamics notwithstanding, 16S ribosomal RNA gene-based studies have revealed recurring diversity patterns within the community of free-living bacteria (bacterioplankton) that respond to phytoplankton blooms [1–6]. Heterotrophic members of the *Bacteroidetes*, *Gammaproteobacteria*, and the alphaproteobacterial *Roseobacter* clade are often among the dominant and recurring responders that partake in the degradation of marine phytoplankton-derived organic matter [7].

A substantial portion of this organic matter consists of polysaccharides that act as algal storage compounds, cell wall components, and exudates. The proportion of these polysaccharides varies depending on algae species and growth stage, and ranges from 13% to ~90% of algal dry-mass [8]. Monosaccharide compositions are often dominated by glucose, mannose, fucose, arabinose, xylose, rhamnose, and galactose (reviewed in [9, 10]). However, linkage types and the overall glycan structures remain mostly elusive as so far only few structures have been resolved [9, 11, 12]. One of the better studied glycans is laminarin, a β-1,3-glucan with occasional 1,6-branches, that acts as a storage compound in brown algae and diatoms (both stramenopiles). Annual production has been estimated to amount to 5–15 Pg (1 Pg × a^−1^ = 10^15^ g × a^−1^) [13], which is equivalent to a third of the world’s oceans’ total annual primary production of 45–50 Pg [14].

Enzymes for the degradation and modification of polysaccharides are widespread among bacterial and archaeal phyla. *Bacteroidetes* have evolved a unique degradation machinery that is usually encoded in so called polysaccharide utilization loci (PULs) [15]. PULs harbor genes that encode degradative Carbohydrate-Active enZymes (CAZymes), such as glycoside hydrolases (GHs), polysaccharide lyases (PLs), or carbohydrate esterases, and accessory proteins, such as proteins carrying carbohydrate-binding domains (CBMs), proteases (targeting glycoproteins), and sulfatases (targeting sulfated polysaccharides). In a concerted effort, extracellular CAZymes degrade polysaccharides into size-ranges that can pass via TonB-dependent outer membrane transporters into the periplasm, where they are protected from competing bacteria and can be further degraded to monosaccharides. These monosaccharides are subsequently taken up across the cytoplasmic membrane by means of dedicated transporters. TonB-dependent uptake is either selfish by taking up all initial cleavage products from the cell surroundings and thereby avoiding cross feeding, as described for α-mannan degradation by a human gut *Bacteroidetes* [16], or semi-selfish, with some cleavage products available to other bacteria [17]. Uptake of polysaccharides into the periplasmic space was recently demonstrated for marine *Bacteroidetes* using fluorescent labeling [18].

The first PUL was identified in the human gut bacterium *Bacteroides thetaiotaomicron* [19] and acts on starch (Sus = starch utilization system). Besides CAZymes, it features a gene tandem coding for SusC and SusD. SusC represents the actual TonB-dependent transporter, and SusD an associated substrate-binding outer membrane lipoprotein [20]. The *B. thetaiotaomicron* Sus locus furthermore features genes coding for the accessory SusEFG proteins. The structure and mechanism of the core SusCD substrate-binding and transport complex was recently resolved in great detail [21], whereas the exact functions of SusEFG are less clear. For SusE, a role as modulator of oligosaccharide uptake size has recently been described [22]. Most PULs in *Bacteroidetes* feature a characteristic *susCD-like* gene tandem, while detectable homologs to *susE* are rare. Until now *susD*-like genes have been found exclusively in *Bacteroidetes*. The efficacy of SusD-mediated substrate binding likely constitutes a major contribution to the *Bacteroidetes’* competitiveness in glycan acquisition.

By now PULs with diverse specificities have been described in *Bacteroidetes* from various habitats (for review see [23]), including metagenomic studies of the fecal microbiome of the North American beaver [24] or the moose rumen [25]. In strains of marine *Bacteroidetes*, PULs have been experimentally verified that target agar/porphyran [26], alginate and laminarin [27–29], carrageenan [30], and ulvan [31, 32]. These studies focused on selected model bacteria, whereas ecological studies that provide a more holistic perspective in terms of environmental relevance are just emerging (e.g. [33–35]).

In previous work, using a series of ten metagenomes from spring phytoplankton blooms in the southern North Sea during the years 2010–2012, we identified fingerprint-like patterns in CAZyme, transporter, and sulfatase gene content within distinct clades of free-living *Bacteroidetes* [4]. We also investigated PUL diversity and in situ expression by sequencing the genomes of 53 isolated strains of North Sea *Flavobacteriia* together with a series of 14 metaproteomes [35]. However, time intervals between individual metagenomes were too wide to capture the rapid changes that were occurring within the bacterioplankton communities [4]. Isolated *Flavobacteriia* strains on the other hand rarely belonged to species that were abundant during the spring algae blooms (e.g. [36]).

In the present study, we therefore added 28 metagenomes to our initial dataset [4], resulting in a dense series of 38 points in time for the consecutive spring blooms of 2010–2012. With recent advances in binning of metagenomes into metagenome-assembled genomes (MAGs; e.g. [37, 38]), we were able to obtain 1286 manually curated *Bacteroidetes* MAGs representing ~120 distinct species. Together with 1815 MAGs that we obtained from other taxa, this dataset provides genomic data of so far unmatched temporal and taxonomic resolution of as yet uncultured algae bloom-associated marine bacterioplankton species, and allowed us to substantially enhance our understanding of the PUL contents of as yet uncultured *Bacteroidetes*.

## Materials and methods

### Sampling

Surface seawater from the long-term ecological research site ‘Kabeltonne’ at Helgoland island in the southern North Sea (54° 11.3′ N, 7° 54.0′ E) was collected during spring phytoplankton blooms from 2009 to 2012. Free-living bacteria were separated from particle-attached bacteria by prefiltration through 10 and 3 µm pore-size filters and collected on 0.2 µm pore-size filters as described elsewhere [4, 39]. DNA for metagenomics and proteins for metaproteomics were subsequently extracted from retained biomass.

### Metagenome sequencing, assembly, and automated binning

Thirty-eight surface seawater metagenome samples were sequenced at the Department of Energy Joint Genome Institute (DOE-JGI, Walnut Creek, CA, USA) as previously described for a subset of ten of these [4] (2010/03/03, 2010/04/08, 2010/05/04, 2010/05/18, 2011/03/24, 2011/04/28, 2011/05/26, 2012/03/08, 2012/04/16, and 2012/05/10). Additional 28 samples were sequenced (2010/03/30, 2010/04/13, 2010/04/20, 2010/04/23, 2010/04/30, 2010/05/11, 2011/03/21, 2011/03/28, 2011/03/31, 2011/04/04, 2011/04/07, 2011/04/14, 2011/04/21, 2011/04/26, 2011/05/06, 2011/05/09, 2011/05/12, 2011/05/16, 2011/05/19, 2011/05/23, 2011/05/30, 2012/04/05, 2012/04/12, 2012/04/26, 2012/05/03, 2012/05/24, 2012/05/31, and 2012/06/07). The latter samples were sequenced on the Illumina HiSeq 2500 platform (Illumina, San Diego, CA, USA) with four samples pooled per flow-cell lane, resulting in about one fourth of reads per sample compared to the initial samples.

Quality filtering and trimming of raw reads, metagenome assembly, initial binning using CONCOCT [40], and subsequent refinement to MAGs was performed as part of the anvi’o pipeline [41] as described in [42]. Details are provided in the Supplementary Text. The resulting MAGs have been submitted to the European Nucleotide Archive under accession PRJEB28156 (see Supplementary Tables S1 and S2).

### Bin phylogenomic analysis, refinement, and reduction of redundancy

Initial binning yielded 6455 bins. CheckM v1.0.7 [43] was used to assess their quality (lineage_wf workflow) and subsequently to place the bins in a reference genome tree (tree_qa command). Bins were selected for refinement, if (i) CheckM placed them within the *Bacteroidetes* and their completeness was >40%, or (ii) if at least one *susD*-like gene was annotated, irrespective of taxonomic placement and completeness values (1185 bins). Bins were refined using the anvi’o anvi-refine command through visually inspecting contig coverage and GC profiles and their positioning after hierarchical clustering. Bins were split when profiles suggested so. This resulted in a set of 1456 refined MAGs, 1286 of which were classified as *Bacteroidetes* (verified by reanalysis with CheckM). In the same manner the remaining initial bins yielded further 1815 MAGs affiliating with other taxa (Supplementary Table S1).

Since metagenomes were assembled and binned separately, redundant MAGs were obtained. Therefore, we used Mash v1.1.1 (default sketch size: 1000; default k-mer size: 21) [44] to cluster the *Bacteroidetes* MAGs into 110 approximate species clusters (henceforth referred to as Mash-clusters—also designated as ‘metagenomic species’ in a recent study on human gut microbiota [45]). Calculated Mash distances have been shown to correlate well with average nucleotide identities (ANI), i.e. a Mash distance of ≤0.05 correlates with ≥95% ANI, a common threshold for genomes to be considered to belong to the same species [46]. Mash-clusters were visualized using Cytoscape v3.5.0 [47].

The maximum number of MAGs per Mash-cluster was 38 (median: 6, mean: 10.5 SD: 9.6). CheckM was used to evaluate the quality of individual Mash-clusters using the *Bacteroidetes*-specific marker set (Supplementary Table S2). Mash-clusters with less than two MAGs of ≥70% completeness (19 of 110) and singleton MAGs ≤70% completeness and contamination ≥10% (90 of 119) were excluded from further analysis. Two representative MAGs (based on highest completeness) were chosen from each of the remaining 91 Mash-clusters and together with the 29 remaining singleton MAGs (≈120 species) their phylogenetic affiliations were analyzed using the GTDB-Tk genome-based taxonomy (GTDB-Tk v0.0.8 with GTDB version 83 [48]). Only closely related sequences to our MAGs were extracted from the GTDB-Tk concatenated reference alignment of 120 bacterial marker genes, and a phylogenetic tree was reconstructed using RAxML v8.2.10 [49] with automatic selection of the substitution model and rapid-bootstrapping with 1000 resamples (-m PROTGAMMAAUTO -p 12345 -x 12345 -# 1000) that was visualized using iTOL [50].

### PUL prediction and SusC/D protein trees

Genes were predicted using Prodigal [51] from within anvi’o. PUL genes were predicted using a combination of HMMER v3.1b2 [52] searches against the dbCAN [53], Pfam [54], and TIGRFAM [55] databases in conjunction with DIAMOND blastp v0.8.27 [56] searches against the CAZy database [57]. Specifically, we used HMMER against the dbCAN v6 models for all CAZy families, the Pfam profile for sulfatases (PF00884), the SusD Pfam models (PF07980, PF12741, PF14322, and PF12771) as also used by PULDB [58, 59] and the TIGRFAM profile for SusC (TIGR04056; OMP_RagA_SusC). HMMER results were filtered with the dbCAN hmmer-scan-parser script, filtering for multiple annotations, *e*-values and a 30% domain coverage minimum. In the second step the HMMER results of all CAZy families were filtered based on whether these proteins had a DIAMOND blastp hit in sensitive mode (-sensitive) with ≥30% identity, at least 40% query coverage and an *e*-value of ≤E−20 against proteins from the same CAZy family in the CAZy database (downloaded from the dbCAN webpage on 2017/07/20). Only these annotations were considered for subsequent PUL prediction.

Potential PULs were extracted by finding all loci, where at least three predicted PUL genes (encoding sulfatases, CAZymes, SusC- or SusD-like proteins) were less than ten genes apart, unless the genes were exclusively glycosyltransferases. Further processing required a PUL to have at least one *susC*- or *susD*-like gene and encode at least two degradative CAZymes from the GH or PL families.

SusC- and SusD-like protein sequences from all predicted PULs (SusC: 1,195; SusD: 1,311) were used for tree calculation. Included were PUL SusC and SusD proteins from isolate genomes [35] and from metaproteomes (see Supplementary Text for details). Multiple sequence alignments were calculated using MAFFT v7.313 [60] with L-INS-I and trees were calculated using FastTree v2.1.10 [61]. SusC and SusD proteins in the tree were clustered at ≥95% nucleotide identity. From these clusters we selected representative sequences with the following criteria: (i) presence in at least four metagenomes, or (ii) presence in three metagenomes, if at the same time expression was indicated by the presence of a closely related sequence (≥90% amino acid identity) in one of the metaproteomes. The resulting datasets of 131 SusC and 130 SusD sequences, representing 910 SusC and 987 SusD sequences from the metagenomes, were used to calculate simplified trees using the same method for SusC/D alignment, but RAxML v8.2.10 with automatic substitution model selection and rapid-bootstrapping with 1000 resamples (-m PROTGAMMAAUTO -p 12345 -x 12345 -# 1000) for tree calculation. Trees were subsequently visualized using iTOL.

Subsequently all representative SusC/Ds and their corresponding PULs were linked to individual Mash-clusters and their taxonomies. A species-level affiliation was only accepted for cases where at least half of the represented SusC/D sequences belonged to a single Mash-cluster. In cases where less than half but still a majority of the represented SusC/D sequences belonged to a single Mash-cluster, the corresponding PULs were considered to be putatively affiliated with the taxonomy of that Mash-cluster. Remaining SusC/D sequences and corresponding PULs belonging to different Mash-clusters of one and the same clade were finally assigned that clade-level taxonomy.

### MAG and PUL abundance estimates

Metagenome reads from all sampling dates were error-corrected with metaSPAdes [62] and subsequently mapped onto singleton MAGs and the two representative MAGs of each Mash-cluster. In the latter case, mean values of the representatives were used as approximation for the Mash-cluster’s abundance. Read mapping and postfiltering of SAM files were performed using the parameters described for metagenome sequencing, assembly, and automated binning (Supplementary Text). Final abundance values were calculated as reads per kilobase per million [RPKM = (number_of_mapped_reads_on_MAG × 1,000,000)/(assembly_length_of_MAG_in_kbp × total_number_of_reads)].

### Metaproteomic bacterioplankton analyses

Metaproteomes were obtained during spring phytoplankton blooms as described in [39] and [35] for the following 14 dates: 2009/03/31, 2009/04/07, 2009/04/14, 2009/04/21, 2010/03/03, 2010/04/08, 2010/05/04, 2010/05/18, 2011/03/24, 2011/04/28, 2011/05/26, 2010/03/08, 2010/04/16, and 2010/05/10 (for details see Supplementary Table S1). Protein extraction from bacterioplankton biomass, separation, and tryptic digestion was carried out as described previously [35]. Fragment detection was carried out using an LTQ Orbitrap Velos mass spectrometer (Thermo Fisher, Bremen, Germany). The spectrometric data has been deposited at the PRIDE repository [63] with the project ID PXD008238 (for details see Supplementary Table S1).

Mass spectrometric data was analyzed using Sequest v27r11 (Thermo Fisher Scientific, San Jose, CA, USA). Searches were carried out against a forward-decoy database of all protein sequences from all corresponding metagenome samples combined (6,194,278 non-redundant sequences) using the uclust option of USEARCH v6.1.544 [64] (options: cluster_fast; nucleotide identity 0.99; maxhits 5; maxrejects 30) and contained 3,212,324 sequences. Common laboratory contaminants were included in all databases. Technical duplicates of each sample were searched together to obtain averaged spectral counts. Validation of protein and peptide identifications was performed with Scaffold v4 (Proteome Software Inc, Portland, OR, USA) using the parameters previously described [39], and normalized spectral abundance factors (%NSAF) were calculated [65] to allow for semiquantitative analyses. The NSAF quantitation measure is commonly used in non-gel-based label-free shotgun proteomics. A %NSAF of 1 corresponds to 1% of all mass-adjusted spectral count data in a given proteome experiment.

## Results

### Phylogeny of *Bacteroidetes* MAGs

We obtained 1286 manually refined MAGs affiliating with the *Bacteroidetes* phylum (Supplementary Table S2). Five hundred and sixty-nine (44%) had a completeness of >90% with <5% contamination, but lacked complete rRNA operons, which rarely assemble in short read shotgun metagenomics. These MAGs hence do not fulfill the strict MIMAGs ‘high-quality’ criteria [66], but they do correspond to the ‘near complete’ category recently used by Almeida et al. [45]. A total of 470 MAGs were ≥50–90% complete with <10% contamination (MIMAGs: ‘medium-quality’), and 218 had a completeness below 50% with <10% contamination (MIMAGs: ‘low-quality’).

**Fig. 1:**
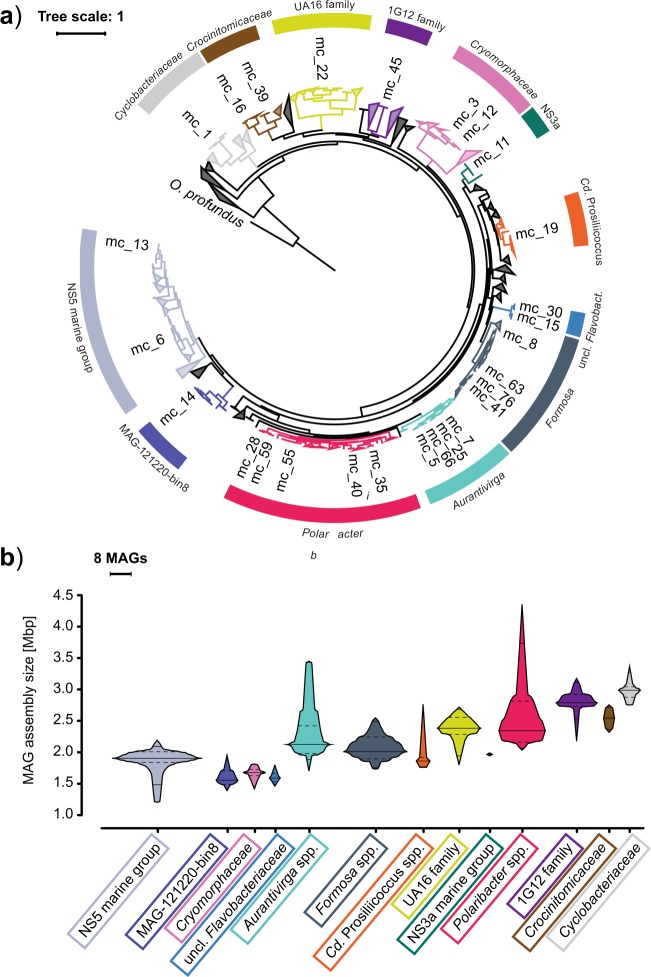
**a** Maximum-likelihood tree of Mash-clusters based on concatenated marker proteins according to the GTDB-Tk genome phylogeny [48]. Only Mash-clusters (mc) were included that contained at least two MAGs ≥70% completeness, plus additional 29 singleton MAGs with ≥70% completeness, and ≤10% contamination. Mash-clusters reaching high abundances (>5 RPKM at one time-point or 38 RPKM at all time-points combined) during the sampling period are highlighted together with their taxonomic affiliations. Scale bar: mean number of amino acid substitutions per site. Outgroup: *Oceanithermus profundus*. **b** Violin plots of MAG assembly size distributions within clades of abundant Mash-clusters (colored areas). Area width corresponds to MAG numbers. Solid lines represent median and deciles, and dashed lines represent quartiles

GTDB-Tk analysis [48] revealed that about 75% of the MAGs belonged to clades that we previously identified as key players during North Sea spring blooms of 2010–2012 [4, 5], including the *Formosa* (GTDB-Tk genus UBA3537), *Polaribacter*, *Aurantivirga* (GTDB-Tk genus SCGC-AA160-P02), *Candidatus* Prosiliicoccus (GTDB-Tk genus HC6-5), the NS3a and NS5 marine groups (GTDB-Tk genera MAG-120531 and MS024-2A), and the GTDB-Tk genus-level clade MAG-121220-bin8, which all belong to the *Flavobacteriaceae* family (Fig. 1a, Supplementary Fig. S1). Other families represented were the *Cryomorphaceae* including the VIS6 clade (GTDB-Tk genus UBA10364), the families UA16, 1G12, and *Crocinitomicaceae* (previously part of the *Cryomorphaceae* family), and the *Cyclobacteriaceae* of the *Cytophagales* order (Fig. 1a, Supplementary Fig. S1). In total 27 multi-MAG Mash-clusters from these 13 *Bacteroidetes* clades were identified as having abundances exceeding five RPKM at a single time-point or 38 RPKM at all time-points combined (2 RPKM ≈ 1% relative abundance detected by fluorescence in situ hybridization [4]).

Among the notable species we detected were members of the genera *Formosa* and *Polaribacter*, both of which reached well above 20% relative abundance during the North Sea spring phytoplankton blooms in 2009 [39] and recurred in 2010–2012 [4]. Mash-clusters 55 and 47 belong to the same species as the strains *Polaribacter* sp. Hel1_33_49 and *Formosa* sp. Hel1_33_131, respectively, which were isolated from the southern North Sea [67, 68]. The latter, however, was not among the abundant Mash-clusters (maximum: 3.5 RPKM). For all other abundant Mash-clusters representing approximate species there are no isolated strains yet.

MAG sizes within the 13 most abundant *Bacteroidetes* clades were mostly in the range of 1.5–3 Mbp, which conforms to the lower range of the *Bacteroidetes* genome size spectrum (e.g. [29]). In particular, MAGs from the NS5 marine group, the MAG-121220-bin8 genus, the *Cryomorphaceae* and two unclassified *Flavobacteriaceae* clades were consistently below 2 Mbp (Fig. 1b, Supplementary Fig. S1). MAGs from the closely related genera *Aurantivirga* and *Polaribacter* exhibited the broadest size spectra, ranging from 1.7 to 3.5 Mbp and 2.1 to 4.3 Mbp, respectively (Fig. 1b, Supplementary Fig. S1).

### Seasonality of *Bacteroidetes* MAGs

**Fig. 2:**
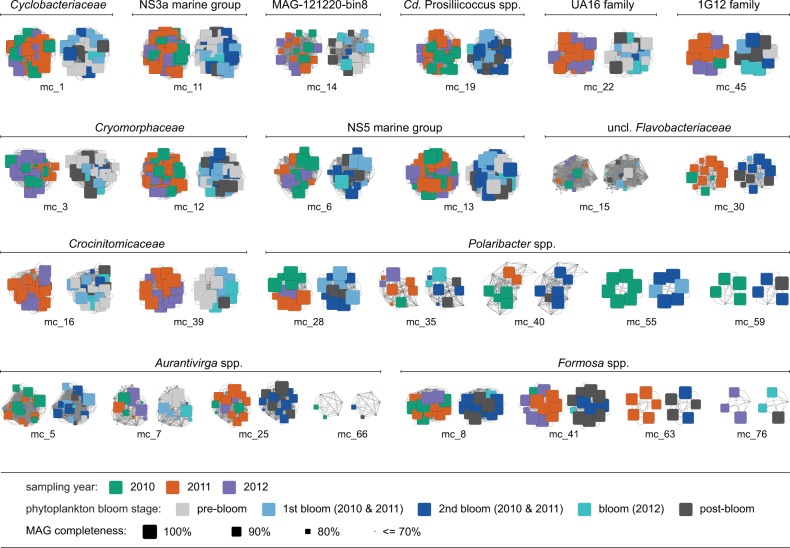
Composition of abundant *Bacteroidetes* Mash-clusters with respect to sampling year (left) and phytoplankton bloom stage (right). Squares represent individual MAGs with sizes corresponding to completeness, while gray lines indicate Mash distances ≤0.05, and thus the approximate species connections for MAGs in each Mash-cluster. Color coding of the left hand side clusters indicates the year in which individual MAGs were retrieved. The majority of Mash-clusters are multi-year clusters as they contain MAGs assembled in all three or at least two years. Color coding of the right hand side clusters represents phytoplankton bloom stages, revealing Mash-clusters predominantly retrieved from metagenomes obtained during phytoplankton blooms, such as *Formosa* spp., *Polaribacter* spp., and *Cd*. Prosiliicoccus spp. Mash-clusters

Based on time-point and chlorophyll *a* concentration we categorized our 2010–2012 metagenomes into pre-bloom, (mid) bloom, and post-bloom periods (Supplementary Fig. S2). The metagenomes of the 2010–2011 bloom periods were further subdivided into primary and secondary blooms, since blooms in these years were bimodal with two extended chlorophyll *a* peaks. Phytoplankton composition differed between spring blooms, with 2010 and 2011 being dominated by different diatom species and in 2012 a less intense and shorter bloom dominated by *Chattonella* species [4]. Nonetheless MAGs of individual Mash-clusters often originated from all three or at least two years (Fig. 2, Supplementary Fig. S3). This corroborates our previous suggestions that a large number of abundant species were recurrent [4, 5]. A few Mash-clusters contained MAGs from almost all sampling time-points, indicating that the corresponding species are autochthonous rather than specialized on bloom situations. In contrast, Mash-clusters of *Formosa* spp., *Polaribacter* spp., and *Cd*. Prosiliicoccus spp. as well as Mash-clusters 6, 25, and 30 displayed high prevalence specifically during bloom and postbloom stages (Fig. 2). *Polaribacter* Mash-clusters 35 and 40 also displayed subclusters of MAGs that were present only in particular years and seasons (Supplementary Fig. S4).

### PULs in *Bacteroidetes* Mash-clusters

**Fig. 3:**
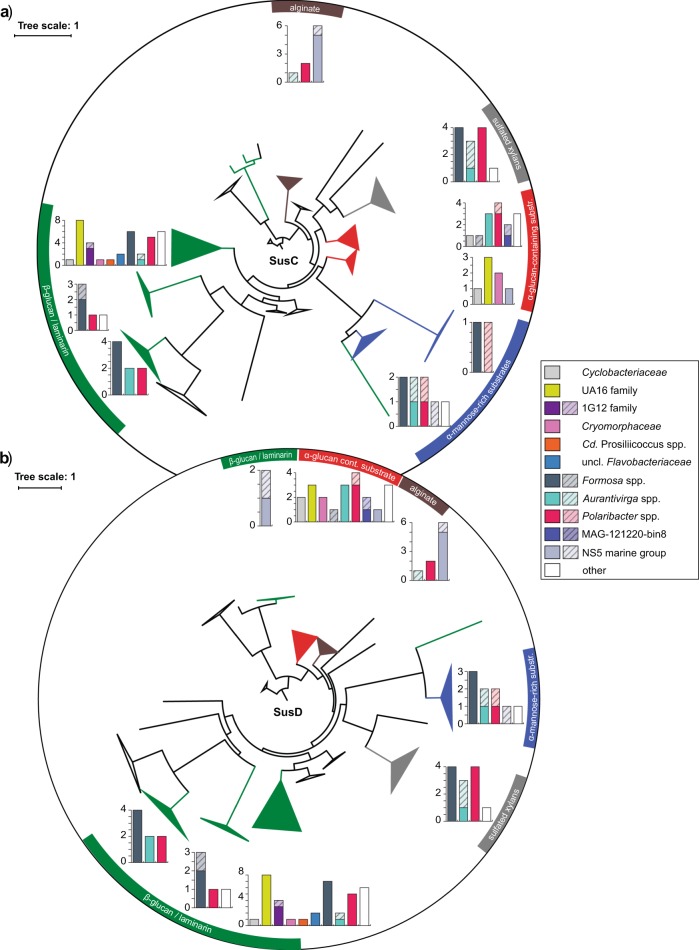
SusC (**a**) and SusD (**b**) tree of all 131 representative SusC-like and 130 SusD-like proteins located within PULs in the metagenome dataset. Protein sequences were aligned using MAFFT L-INS-I [60] and trees calculated using RAxML [49]. Branches were collapsed and color coded based on substrate predictions derived from CAZyme analysis of the respective PULs [35]. Bar plots indicate taxonomic affiliation of respective PUL contigs for each collapsed branch. Block colors indicate SusC and SusD proteins whose corresponding contigs were consistently binned into a single Mash-cluster, and hatched areas indicate binning consistent on genus level

We identified 116 representative PUL-containing contigs with a total of 131 *susC* and 130 *susD* genes (Supplementary Data). Some contigs carried two or three *susC/D* pairs or even two distinct PULs. Seventy-eight percent of these *susC/D* pairs could be assigned to specific Mash-clusters and 17% putatively to specific Mash-clusters or a specific genus. Genome trees of corresponding protein sequences showed clustering based on predicted PUL substrate specificity rather than a taxonomic signal, as we have previously described [35]. Substrate predictions suggested five major polysaccharide categories that free-living *Bacteroidetes* targeted during the investigated North Sea spring phytoplankton blooms (Fig. 3).

#### Beta-glucans/laminarin

**Fig. 4:**
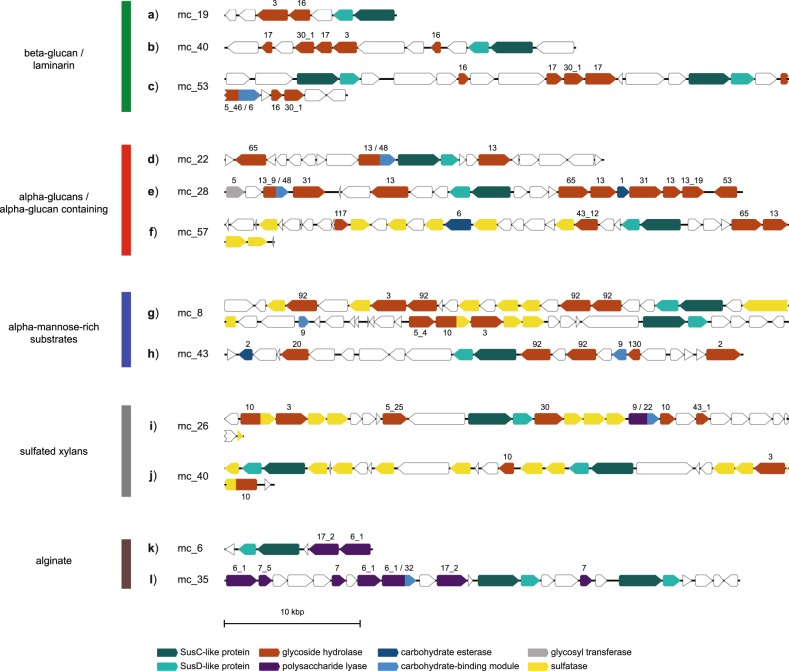
Polysaccharide utilization loci representing common detected PUL patterns. Mash-cluster affiliations for each PUL-contig are indicated. Color coding of genes indicate gene types and corresponding numbers indicate CAZyme family associations

Predicted β-glucan PULs were found for many of the abundant *Bacteroidetes* genera, most of which presumably target laminarin. SusC/D sequences from these PULs clustered in respective SusC/D trees in a single clade with only few exceptions, e.g. the SusC/D sequences of β-glucan PULs from the NS5 marine group that clustered separately (Fig. 3). The main cluster comprised sequences from 37 predicted laminarin PULs attributed to at least ten genera. These PULs could be subdivided into three variants, all of which carried a GH16 gene (Fig. 4a–c). The shortest variant showed a combination of GH3 and GH16 enzymes, sometimes accompanied by a GH2 or a GH30_1 (Fig. 4a; variant A in [35]). This variant was present in several Mash-clusters attributed to the *Formosa*, *Cd*. Prosiliicoccus, and *Polaribacter* genera, Mash-clusters 15 and 30 (uncl. *Flavobacteriaceae*), and the families UA16, 1G12, and *Cryomorphaceae*.

A second variant with the combination of GH16, GH30_1, and often two GH17 enzymes (Fig. 4b, variant B in [35]) was mainly present in *Polaribacter* and *Aurantivirga*. Other less abundant genera carrying this variant were Mash-clusters 9 (*Algibacter*-related), 10 (genus UBA6710), 34 (genus UBA1994), and 70 (unclassified on genus level).

The third variant contained a combination of a CBM6-containing GH5_46 and a GH16 enzyme (variant C in [35]). This PUL type has so far not been experimentally verified as targeting laminarin, but consistent clustering of the respective SusC/D sequences with verified laminarin PULs suggests laminarin or β-glucans as substrates. Automatically predicted PULs from the PULDB often show these enzymes in combination with either a GH30 or a GH3. This PUL type in the large laminarin cluster was restricted to the VIS6 *Cryomorphaceae* and the UA16 genus within the UA16 family.

PULs similar to this third variant were present in separate clusters in the SusC/D tree, and belonged mostly to *Formosa*, *Polaribacter* or *Aurantivirga* Mash-clusters. These PULs code for a combination of GH5_46, GH30_1 and GH16, though the GH16 was missing in some of the *Formosa* PULs. At least three such PULs were located on contigs with two *susC/D* pairs (Fig. 4c)—one pair of which clustered in the large laminarin cluster. To clarify whether these PULs are co-regulated, target different laminarin types or increase each other’s efficiency can only be tested experimentally with isolate strains carrying such a PUL combination.

#### Alpha-glucan-containing substrates

Larger clusters were also observed for PULs related to the degradation of α-glucans. The SusD tree contained one and the SusC tree two such clusters. With two exceptions, the corresponding PULs were characterized by GH13 family genes, which are known to act on α-glucose. These PULs can be separated into rather simple variants containing at most four to five CAZymes and more complex and variable PULs, some of which contain sulfatases. The simpler PULs were prevalent within the VIS6 *Cryomorphaceae* and the UA16 families and contained a GH65 and one to two GH13 genes (Fig. 4d). The latter sometimes had a CBM48, a carbohydrate-binding module known to bind α-glucans. The GH13 plus GH65 combination has already been described in a PUL from *Gramella forsetii* KT0803^T^ as targeting α-1,4-glucans [28]. The GH13 family, among other functions, contains α-amylases that act on amylose of starch or glycogen. The GH65 family contains maltose phosphorylases that cleave maltose to glucose and glucose-1-phosphate.

The more complex PULs carried several GH13 and often a GH65 gene, additionally often a GH43_12 gene and sometimes sulfatases (Fig. 4e, f). The substrate of these PULs remains unclear, but the similarity in SusC and SusD protein sequences and the presence of GH13 enzymes suggest that the oligosaccharide taken up is similar in structure compared to those originating from the initial breakdown of simple α-glucans. In terms of taxonomy these more complex PULs could be detected in clades such as the *Cyclobacteriaceae*, the NS5 marine group, *Formosa*, and the MAG-121220-bin8 genus.

#### Alpha-mannose-rich substrates

SusC and SusD sequences from GH92-rich PULs also formed coherent clusters. GH92 family proteins are exo-acting α-mannosidases that cleave different linkage types (e.g. [69]). Some but not all of these PULs also contained a larger number of sulfatase genes.

Sulfatase-containing GH92-rich PULs harbored three to four GH92 genes with up to six sulfatase genes and additional GHs of families GH2, GH3, and GH43_2 (Fig. 4g). These PULs affiliated with *Formosa*, *Polaribacter*, *Aurantivirga*, and an *Algibacter*-related Mash-cluster (mc_9). Similar types have already been reported for *Polaribacter* and *Formosa* species [29, 33, 35] and were hypothesized to target glucuronomannan substrates [35]. Such a polysaccharide was recently detected in the cell wall of the diatom *Phaeodactylum tricornutum*, and has been described as a linear poly-α-1,3-mannan decorated with sulfate ester and β-D-glucuronic residues [11].

Sulfatase-free GH92-rich PULs contained a combination of GH92, GH130, GH20, and GH18 genes and sometimes a CBM9-containing, GH3 or GH2 gene (Fig. 4h). Often a putative CE2 was also encoded. Similar PULs have been predicted in *Flavobacterium* sp. SCGC AAA160-P02 (*Aurantivirga*) and various *Salegentibacter* species in PULDB. It is noteworthy that similar PULs have been detected on *Flavobacteriia* fosmids from the North Atlantic, which have been hypothesized to target mixed-linkage glucans including enzymes involved in xylan metabolism (CE2 acetyl-xylan esterase and CBM9) and degradation of mannose-rich substrates (GH92 and GH130) [34]. The sulfatase-free GH92 PULs in our study were distributed among *Formosa*, *Polaribacter*, and *Aurantivirga* genera and the NS5 marine group.

#### Sulfated xylans/xylose-rich substrates

PULs putatively targeting sulfated xylans or xylose-rich substrates exhibited two conserved patterns. The first included a sulfatase domain-containing GH10, a GH3 and two additional sulfatase genes (Fig. 4i, j). The second harbored a GH30, three sulfatases, a PL9 with a CBM22, a GH10 and a GH43_1 gene (Fig. 4i). These two patterns were sometimes combined in a single PUL. Known GH10 enzymes are mostly endo-β-xylanases. Families GH3, 30, and 43_1 have a broader substrate spectrum, but all include β-xylosidases. These two types affiliated with *Formosa*, *Aurantivirga*, and *Polaribacter* MAGs and have already been described to be present in marine *Bacteroidetes* [34, 35].

#### Alginate

The alginate PUL cluster was dominated by NS5 marine group Mash-clusters, though also two *Polaribacter* and one *Aurantivirga* PUL were present. The alginate PULs contained varying combinations of PL6, PL7, and PL17 alginate lyase genes (Fig. 4k, l). Such a PUL has been described in *G. forsetii* KT0803^T^, and is also found in numerous other marine *Flavobacteriia* (e.g. [35]).

### Mash-cluster PUL repertoires and abundance patterns

**Fig. 5:**
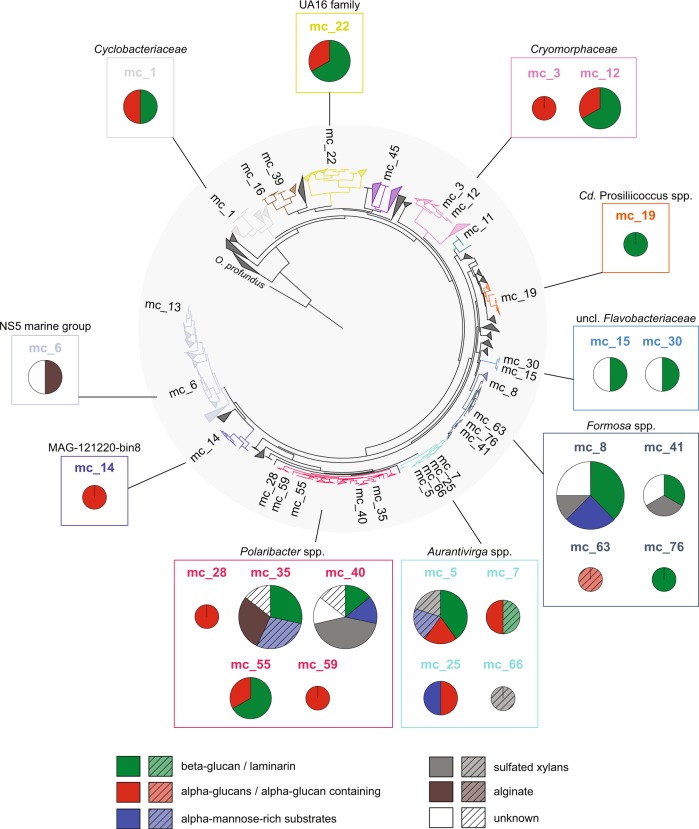
PUL repertoires of abundant Mash-clusters. Pie charts depict the predicted PUL substrates for each Mash-cluster and are sized according to PUL numbers (minimum: 1; maximum: 8 in mc_8). Mash-clusters that lack predicted PULs are not visualized. Box colors indicate PULs consistently binned in the same Mash-cluster, while hatched colors indicate putative PULs present in respective Mash-clusters (majority, but less than 50% of the same SusC/D binned into Mash-cluster)

PUL numbers per individual Mash-cluster ranged from zero to eight with the applied filtering criteria (Fig. 5). Mash-clusters with the broadest PUL spectra belonged to the *Aurantivirga*, *Formosa*, and *Polaribacter* clades. All other Mash-clusters contained at most three PULs, most of which were targeting α- or β-glucans. This separation of clades into narrower vs. broader PUL spectra was also reflected in the individual clade’s abundance patterns.

**Fig. 6:**
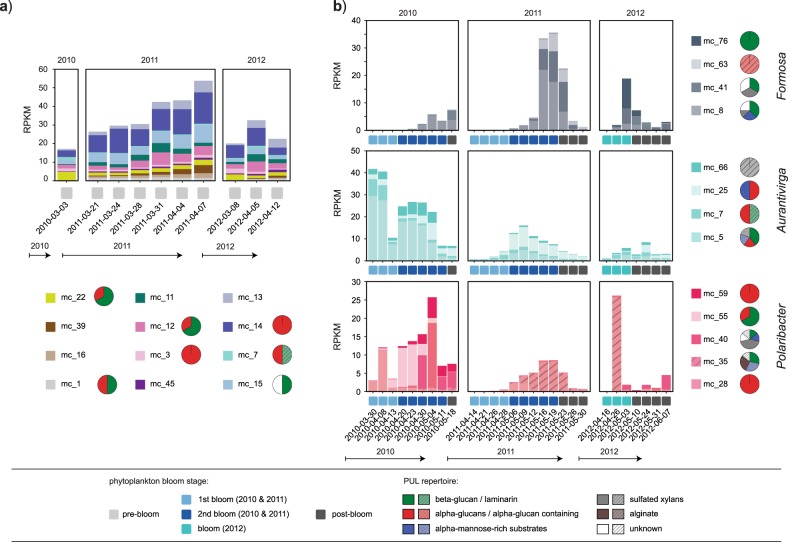
Abundance patterns of Mash-clusters at **a** pre-bloom and **b** (mid-) bloom and post-bloom phytoplankton bloom stages. Mash-cluster abundances are shown as reads per kilobase per million (RPKM) values in all bar charts. **a** All Mash-clusters with ≥1 RPKM at at least one pre-bloom time-point and **b** the abundant *Formosa*, *Polaribacter*, and *Aurantivirga* Mash-clusters. Pie charts depict predicted PUL repertoires of respective Mash-clusters. Abundance patterns of other abundant Mash-clusters are shown in Supplementary Fig. S5, and details on the metagenome datasets are provided in Supplementary Table S1

The spring bloom *Bacteroidetes* community at Helgoland showed a clear shift from a relatively stable low diversity pre-bloom community with few PULs towards a more dynamic and diverse community with more diverse PULs during mid-blooms. In all three years the pre-bloom community was dominated by clades with streamlined PUL repertoires. Mash-clusters reaching at least ≥1 RPKM at pre-bloom time-points carried between zero and three PULs, the majority of which targeted α- or β-glucans (Fig. 6a). Mash-clusters 12 (*Cryomorphaceae*), 14 (MAG-121220-bin8), and 15 (unclassified *Flavobacteriaceae*) were among the *Bacteroidetes* with the highest pre-bloom abundances, reaching up to 17 RPKM (~8.5% relative abundance). Mash-clusters 12 and 14 carried α-glucan PULs and Mash-clusters 12 and 15 carried β-glucan PULs (Figs. 5, 6). Overall, PUL repertoires at pre-bloom time-points indicated that the pre-bloom *Bacteroidetes* community predominantly targeted rather simple glycans.

The mid-bloom bacterioplankton response was more variable in all three years, though often species of the same clades reached high relative abundances. The PUL repertoires of mid-bloom *Bacteroidetes* Mash-clusters were broader compared to those that dominated pre-bloom phases. In the initial mid-bloom phase of 2010 we detected few highly abundant species. Mash-clusters 5 (*Aurantivirga*), 12 (*Cryomorphaceae*), 15 (unclassified *Flavobacteriaceae*), 19 (*Cd*. Prosiliicoccus), and 28 (*Polaribacter*) reached abundances well above 10 RPKM (~5% relative abundance; Fig. 6, Supplementary Fig. S5). All of these Mash-clusters carried PULs, targeting either α-glucans (mc_5, mc_12, and mc_28) or laminarin/β-glucans (mc_5, mc_12, mc_15, and mc_19). In addition, Mash-cluster 5 putatively targets mannose-rich substrates and sulfated xylans. These Mash-clusters, except for mc_5, leveled off in relative abundance towards the next stage of the 2010 bloom, where Mash-cluster 55 (*Polaribacter*) reached abundances of about 12 RPKM. Mash-cluster 55 carried PULs for both β-glucans and α-glucans. Towards the end of the bloom at the last three sampling time-points, Mash-clusters 8 (*Formosa*) and 40 (*Polaribacter*) became abundant. Both showed similar PUL repertoires, as both encoded PULs targeting β-glucans, mannose-rich substrates, and sulfated xylans, as well as unknown substrates.

The 2011 phytoplankton bloom was less intense than in 2010 and so was the bacterioplankton’s response [4]. For example, the response of *Cd*. Prosiliicoccus (mc_19), *Polaribacter*, and *Aurantivirga* was less intense than in 2010 (Fig. 6, Supplementary Fig. S5). Furthermore, the species composition of the *Polaribacter* and *Aurantivirga* clades was different. The *Aurantivirga* clade reached a maximum of 20 RPKM (~10% relative abundance) with Mash-clusters 5 and 25 contributing about equally. The *Polaribacter* clade reached only up to 10 RPKM and was dominated by Mash-cluster 35, which had a broad PUL repertoire targeting β-glucans, putative mannose-rich substrates and alginates (Figs. 5, 6). In contrast, *Formosa* species dominated the *Bacteroidetes* community and reached up to 35 RPKM towards the end of the secondary bloom. Mash-clusters 8, 41, and 63 contributed to the abundances, although they showed different PUL repertoires (Figs. 5, 6).

In 2012 both the overall phytoplankton bloom and the *Bacteroidetes* response were even less intense [4]. Only Mash-cluster 35 (*Polaribacter*) and the *Formosa* clade reached RKPM values above ten and only at two time-points (Fig. 6). There, *Formosa* was dominated by Mash-clusters 41 and 76, both of which carried β-glucan PULs with mc_41 additionally targeting sulfated xylans and as yet unknown substrates.

Overall *Aurantivirga*, *Cd*. Prosiliicoccus, *Formosa*, and *Polaribacter* Mash-clusters were absent, or present at only low abundances (*Aurantivirga)* during the pre-bloom time-points but reached high abundances during the blooms. Thus, these clades constitute major bloom responders [4]. With the exception of *Cd*. Prosiliicoccus [42] these clades featured broad PUL repertoires. *Formosa* and *Aurantivirga* harbored PULs for four of the major substrates targeted by *Bacteroidetes* during phytoplankton spring blooms and *Polaribacter* all five including alginate.

### PUL expression

**Fig. 7:**
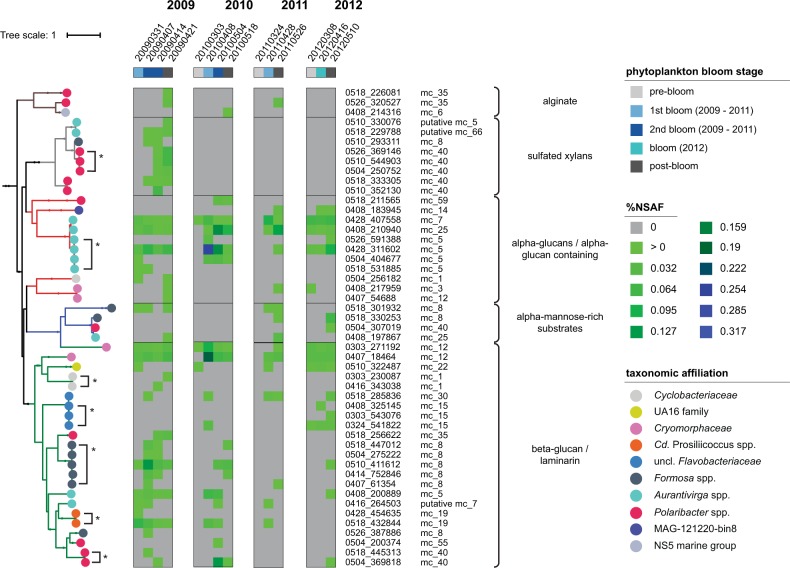
SusC tree of expressed SusC-like proteins matching to PULs of abundant *Bacteroidetes* species. Protein sequences were aligned using MAFFT L-INS-I [60] and trees calculated using RAxML [49]. Substrate predictions for related PULs are indicated by branch colors and vertical brackets to the right hand side of the graph. The heat map indicates expression levels as Normalized Spectral Abundance Factors (%NSAF) for four consecutive blooms (2009–2012). Taxonomic affiliations of respective expressed proteins are shown as colored circles and are additionally indicated by the Mash-cluster affiliation (mc_x). Expressed proteins with an amino acid sequence identity of >95% that are linked to the same PUL type and Mash-cluster are marked with an asterisk. Details on the metaproteome datasets are provided in Supplementary Table S1

Components of TonB-dependent transporters are among the proteins with the highest expression in metaproteomes of phytoplankton-associated bacterioplankton. For example, in earlier studies they accounted for 7% [70] and 13% [39] of the total proteins. SusC/D-like proteins are among these highly expressed TonB-dependent transporters and are suitable as proxies for overall PUL expression [35]. Analysis of SusC sequences from all 14 metaproteomes suggested expression of PULs targeting all five predicted major substrate classes (Fig. 7, Supplementary Table S3; for SusD see Supplementary Table S4). Particularly strong expression was indicated for putative α-glucan PULs in *Aurantivirga* members and for putative β-glucan/laminarin PULs in *Cryomorphaceae* members.

The 14 metaproteomes provided less time resolution than the 38 metagenomes, but still some trends could be observed. For instance, expression of putative sulfated xylan PULs was only indicated in 2009 during the second and post-bloom phases in *Polaribacter* and *Aurantivirga* members, and expression of putative alginate PULs was detected in 2009–2011 only in post-bloom phases. In contrast, putative α- and β-glucan PUL expression was indicated throughout all bloom stages in all years.

## Discussion

Marine *Bacteroidetes* have been regularly observed as major responders during phytoplankton blooms and usually outcompete other bacterial clades during early bloom phases (e.g. [6, 71, 72]). The ability to decompose high-molecular-weight organic matter [73] using PULs with efficient SusC/D-like uptake systems is likely pivotal for the competitiveness of many of these *Bacteroidetes*. Plenty of PULs have been detected in isolated *Bacteroidetes* strains (e.g. [28, 35, 74, 75]). However, many of these are associated with macroalgae (e.g. [76]), whereas only a few are representatives of abundant free-living *Bacteroidetes* during microalgae blooms [29, 35, 36]. Therefore, PUL repertoires of most isolates do not indicate which PULs predominate during such blooms.

Recent improvements in metagenome sequencing, assembly, and binning have enabled large-scale retrieval of PULs from natural bacterioplankton and subsequent linkage to distinct species. This approach works particularly well for bacterioplankton during early spring bloom stages, where there is low evenness due to stark proliferation of few well adapted species, some of which are almost clonal (e.g. [77]).

Our analysis of a dense time series of deeply sequenced bacterioplankton metagenomes in combination with state-of-the-art binning and subsequent PUL prediction and annotation circumvents the isolation problem. However, it also entails a certain margin of error, as PUL functions are predicted based on similarity searches against reference databases and not on laboratory-based experiments with dedicated bacterial strains. Likewise, the sheer size of the dataset (38 metagenomes; 9.9 Gbp of contigs >2.5 kbp) required a rather rigid automated PUL prediction, which neglected noncanonical PULs devoid of a *susC/D* gene pair. It has been shown that some PULs have *susC/D* pairs that are separated from the corresponding CAZyme genes elsewhere in the genome (e.g. [30]). Likewise, PULs from sparsely occurring Mash-clusters were excluded as we restricted our analysis to PULs that occurred in at least four metagenomes. Still the Mash-clusters we describe provide first time insights into the most prevalent PULs and their predicted algal polysaccharide targets within the largely uncultivated planktonic *Bacteroidetes* community during spring phytoplankton blooms in the southern North Sea.

The analyzed *Bacteroidetes* communities were dominated by a few clades, such as *Cd*. Prosiliicoccus, *Formosa*, *Polaribacter*, and *Aurantivirga*. Mash-cluster analysis demonstrated recurrence of these clades in 2010–2012 with some interannual variability, substantiating an initial analysis of a much smaller subset of the metagenomes [4]. This recurrence suggests a high level of specialization on bloom events. Rather small genome sizes with limited numbers of PULs enable these *Bacteroidetes* to quickly respond to phytoplankton blooms with fast growth rates, while targeting only specific subsets of the glycans that algae produce. The average genome size of our Mash-cluster representatives was about 1.5 Mbp lower compared to genome sizes of isolated North Sea strains of 3.83 Mbp [35]. Two published single cell genomes of the NS5 and NS3a marine groups (*Flavobacteria* bacterium MS024-2A; *Flavobacteria* bacterium MS024-3C) are closely related to the mc_13 and mc_11 in our study. These two species feature small genomes (~2 Mbp) and ‘narrow ecological niches’ [78]. It has been speculated that this might be the reason why they resist cultivation, even though they are abundant and widespread [78]. Considering that genome streamlining entails a reduction of physiological flexibility [79], it is not unexpected that a lot of free-living marine *Bacteroidetes* resist conventional isolation techniques.

The majority of abundant PULs that we describe constitute a subset of the PUL spectrum that has so far been described in isolated *Bacteroidetes* strains [29, 35, 36]. These PULs are limited to five major substrate classes of which β-glucans/laminarin and α-glucan-containing substrates are both most abundant as well as present throughout all bloom periods. PULs targeting these simple glycans represent more than half of the described metagenomic PULs (50/131 β-glucans/laminarin; 22/131 α-glucan-containing substrates), which is substantially more than compared to only about 25% of PULs in the genomes of isolated North Sea *Bacteroidetes* strains [35]. This suggests that both β-glucans/laminarin and α-glucan-containing substrates make up the majority of substrates available to the bacterial community during phytoplankton blooms, which is corroborated by our metaproteome data. Laminarin is the major storage compound of diatoms and so are slightly different types of β-glucans in other phytoplankters [80]. Alpha-glucans on the other hand act as storage compound in bacterial and animal cells (e.g. glycogen). Thus, laminarin and α-glucans are constantly processed and released during bloom events due to factors such as grazing, viral lysis, or autologous cell death that influence microbial mortality (reviewed in [81]).

We observed an average of only 2.2 PULs per Mash-cluster compared to an average of 7.5 PULs for genomes of isolated North Sea *Bacteroidetes* strains [35], and similar numbers in other marine *Bacteroidetes* (e.g. [76]). Notable exceptions were the *Polaribacter*, *Formosa*, and *Aurantivirga* Mash-clusters, with some species exhibiting the highest PUL numbers (maximum: eight) and most diverse predicted substrate spectra. These clades all share the potential to degrade α-mannose-rich substrates and sulfated xylans/xylose-rich substrates. The coeluting sugars mannose and xylose were detected as the second most abundant monosaccharides in the total combined carbohydrates during the 2010 bloom [82]. Still, the glycan niches of the most abundant bacteroidetal bacterioplankters are rather narrow, which is why the remineralization of algal glycans is a concerted effort of many of these clades.

Throughout the entire time series some clades featured rather constant PUL repertoires, e.g. *Cyclobacteriaceae*, whereas there was considerable compositional change in others, e.g. within the broad *Polaribacter* clade. We observed a clear dominance of PULs targeting simple glycans (β-glucans/laminarin and α-glucan-containing substrates) in pre-bloom communities, whereas amidst blooms more complex polysaccharides were targeted as well. We hypothesize that this is the result of two effects. First, bacteria will in general prefer easily degradable substrates such as simple storage glycans over biochemically more demanding ones. Second, the availability of more complex polysaccharides increases over a blooms’ course due to increasing algae mortality rates. For example the proteome data for sulfated xylans suggests this for the year 2009. However, due to the limited time resolution of available metaproteomes (only 14 from 2009 to 2012), no clear temporal patterns in PUL expression could be observed.

It is still an open question which selective effects favor one of two species with similar PUL repertoires over the other. Species with similar PUL repertoires might still prefer different polysaccharides. For example, cocultivation of two gut *Bacteroidetes* suggested that their glycan preferences are genetically hard-wired [83]. In a similar fashion, slight subtle differences in polysaccharide composition might also be a contributing factor. High-resolution PUL in situ expression data over the entire course of a phytoplankton bloom will be necessary to further enhance our understanding, as abundance does not necessarily equate to high activity (e.g. [84]).

The compositional dynamics of phyto- and bacterioplankton communities during blooms are complex, as is the resulting glycan turnover biochemistry. Nonetheless, a limited number of bacterioplankton clades prevail during bloom conditions that carry a limited number of abundant PULs, which in turn target a limited number of major glycan substrates. This means that the attainment of a fundamental understanding of the bulk of glycan-mediated carbon flow during phytoplankton bloom events is within reach.

## Supplementary information

Supplementary Text

Supplementary Figure S1

Supplementary Figure S2

Supplementary Figure S3

Supplementary Figure S4

Supplementary Figure S5

Supplementary Table S1

Supplementary Table S2

Supplementary Table S3

Supplementary Table S4

Dataset 1
