# Supplementary material for: In marine Bacteroidetes the bulk of glycan degradation during algae blooms is mediated by few clades using a restricted set of genes
Source: ISME J. 2019 Jul 17;13(11):2800–16. doi: 10.1038/s41396-019-0476-y (PMC6794258; doi:10.1038/s41396-019-0476-y)
Supplement: Supplementary file 1 — Supplementary Text [file 41396_2019_476_MOESM1_ESM.docx]

***The ISME Journal - Supplementary Text***

**In marine *Bacteroidetes* the bulk of algal glycan degradation during algae blooms is mediated by a few clades using a restricted set of genes**

Karen Krüger^1^, Meghan Chafee^1^, T. Ben Francis^1^, Tijana Glavina del Rio^2^, Dörte Becher^3^, Thomas Schweder^4,5^, Rudolf I. Amann^1*^, Hanno Teeling^1*^

^1^ Max Planck Institute for Marine Microbiology, Celsiusstraße 1, 28359 Bremen, Germany

^2^ DOE Joint Genome Institute, 2800 Mitchell Drive, Walnut Creek, CA 94598, USA

^3^ Institute for Microbiology, University Greifswald, Felix-Hausdorff-Straße 8, 17489 Greifswald, Germany

^4^ Pharmaceutical Biotechnology, Institute of Pharmacy, University Greifswald, Felix-Hausdorff-Straße 3, 17487 Greifswald, Germany

^5^ Institute of Marine Biotechnology, Walther-Rathenau-Straße 49a, 17489 Greifswald, Germany

^*^ Corresponding authors:

Hanno Teeling, Max Planck Institute for Marine Microbiology, Celsiusstraße 1, 28359 Bremen, Germany, email: [hteeling@mpi-bremen.de](mailto:hteeling@mpi-bremen.de), phone: +49 421 2028 976

Rudolf I. Amann, Max Planck Institute for Marine Microbiology, Celsiusstraße 1, 28359 Bremen, Germany, email: [ramann@mpi-bremen.de](mailto:ramann@mpi-bremen.de), phone: +49 421 2028 930

**Running title:** *Bacteroidetes'* glycan usage during algae blooms

**This Supplementary Information PDF file contains:**

- Supplementary Materials and Methods and Results with references

Supplementary Tables are available as separate Excel files.

**Supplementary Materials and Methods**

*Metagenome quality filtering, assembly and automated binning*

Quality filtering and trimming of raw reads, metagenome assembly and binning using CONCOCT (1) and anvi’o was performed as described previously (2). In brief, BBDuk v.35.14 (<http://bbtools.jgi.doe.gov>) was used to remove TruSeq adapters and low quality reads (options: ktrim=r, k=28, mink=12, hdist=1, tbo=t, tpe=t, qtrim=rl, trimq=20, minlength=100). Metagenomic datasets were assembled individually using metaSPAdes v3.10.0 (3) with kmer lengths of 21, 33, 55, 77, 99 and read-error correction enabled. Contigs below 2.5 kbp were excluded from further analyses. Separate binning of contigs from each assembly was performed using CONCOCT (1) integrated in the anvi'o metagenomic workflow (4). Default settings were used during the anvio profiling and merging steps. Read coverage profiles were generated by mapping SPAdes error-corrected reads of the respective sampling date and four additional SPAdes error-corrected read sets from metagenomic samples of the same year to contigs ≥2.5 kbp (Supplementary Table S1). BBMap v35.14 (<http://bbtools.jgi.doe.gov>) was used for read mapping in fast mode, with a minimum mapping identity (minid) of 0.99 and identity filter (idfilter) of 0.97. Subsequently sequence alignment map files were converted to binary format, filtered, sorted and indexed using SAMtools v1.2 (5). Finally, sequence alignment map files were filtered: unmapped reads (-F 4), reads mapped with low quality <10 (-q) and reads found to be PCR duplicates (VALIDATION_STRINGENCY=LENIENT) were removed using Picard tools v1.133 (<http://broadinstitute.github.io/picard>).

*Metaproteome SusC and SusD extraction*

SusC and SusD sequences were extracted from metaproteome data by searching all identified expressed protein sequences (total: 23 917) with Hidden Markov Models, namely the TIGRFAM profile for SusC/RagA (TIGR04056) and all four Pfam profiles for SusD (PF07980, PF12741, PF14322, PF12771). Hits were post-filtered using the dbCAN (6) hmmer-scan-parser script with omission of the filtering step that excludes hits with less than 30% model coverage. All identified SusC/SusD proteins were subsequently integrated in the SusC- and SusD-protein trees and considered as representing *in situ* PUL expression, if they exhibited ≥90% amino acid similarity to any of the metagenome-derived SusC and SusD sequences, respectively (Supplementary Table S3, Supplementary Table S4).

**Supplementary Results**

*PULs in* Bacteroidetes *Mash-clusters*

- *Unknown substrates*

The SusC/D protein trees showed a cluster close to the laminarin/β-glucan cluster, whose corresponding PULs had a combination of CAZymes that is not described in literature so far. These PULs encode similar CAZyme families as laminarin PULs of variant two (3x GH17, GH30_1), but feature a GH92 instead of a GH16 gene. Other PULs in this cluster contained three GH92 genes and either a GH2 or two GH3 genes. A similar PUL has been predicted by PULDB for e.g. *Flavobacterium johnsoniae* UW101, where instead of a third GH92, a GH125 gene is present that has exo-α-1,6-mannosidase activity as only known activity (7). These PULs occurred in both the *Formosa* genus and Mash-clusters 15 and 30 (unclassified *Flavobacteriaceae*), but substrate specificity remains as yet unclear.

Mash-cluster 57 contained the longest PUL in the entire dataset. This PUL harbored two *susC/D* pairs (one with two *susC* homologues), and comprised 20 CAZyme and sulfatase genes. Mash-cluster 57 is a lowly abundant member of the NS5 marine group and was binned from post-bloom time-points in 2011 and 2012 (Supplementary Figure S3). The PUL’s CAZyme composition suggests a sulfated mixed-glucan containing rhamnose, galactose and mannose. The PUL encodes a GH92 (exo-α-mannosidase activity) and several glycoside hydrolase families with enzymes acting on rhamnose or rhamnose-galactose containing substrates, such as two GH105, a GH78 and a GH28.

**References**

1. Alneberg J, Bjarnason BS, de Bruijn I, Schirmer M, Quick J, Ijaz UZ, et al. Binning metagenomic contigs by coverage and composition. Nature Methods. 2014;11(11):1144-6.

2. Francis TB, Krüger K, Fuchs BM, Teeling H, Amann RI. *Candidatus* Prosiliicoccus vernus, a spring phytoplankton bloom associated member of the *Flavobacteriaceae*. Systematic and Applied Microbiology. 2019;42(1):41-53.

3. Nurk S, Meleshko D, Korobeynikov A, Pevzner PA. metaSPAdes: a new versatile metagenomic assembler. Genome Research. 2017;27(5):824-34.

4. Eren AM, Esen ÖC, Quince C, Vineis JH, Morrison HG, Sogin ML, et al. Anvi’o: an advanced analysis and visualization platform for ‘omics data. PeerJ. 2015;3:e1319.

5. Li H, Handsaker B, Wysoker A, Fennell T, Ruan J, Homer N, et al. The Sequence Alignment/Map format and SAMtools. Bioinformatics. 2009;25(16):2078-9.

6. Yin Y, Mao X, Yang J, Chen X, Mao F, Xu Y. dbCAN: a web resource for automated carbohydrate-active enzyme annotation. Nucleic Acids Research. 2012;40(W1):W445-W51.

7. Gregg KJ, Zandberg WF, Hehemann JH, Whitworth GE, Deng L, Vocadlo DJ, et al. Analysis of a new family of widely distributed metal-independent alpha-mannosidases provides unique insight into the processing of N-linked glycans. Journal of Biological Chemistry. 2011;286(17):15586-96.
