# Supplementary material for: In marine Bacteroidetes the bulk of glycan degradation during algae blooms is mediated by few clades using a restricted set of genes
Source: ISME J. 2019 Jul 17;13(11):2800–16. doi: 10.1038/s41396-019-0476-y (PMC6794258; doi:10.1038/s41396-019-0476-y)
Supplement: Supplementary file 2 — Supplementary Figure S1 [file 41396_2019_476_MOESM2_ESM.pdf]

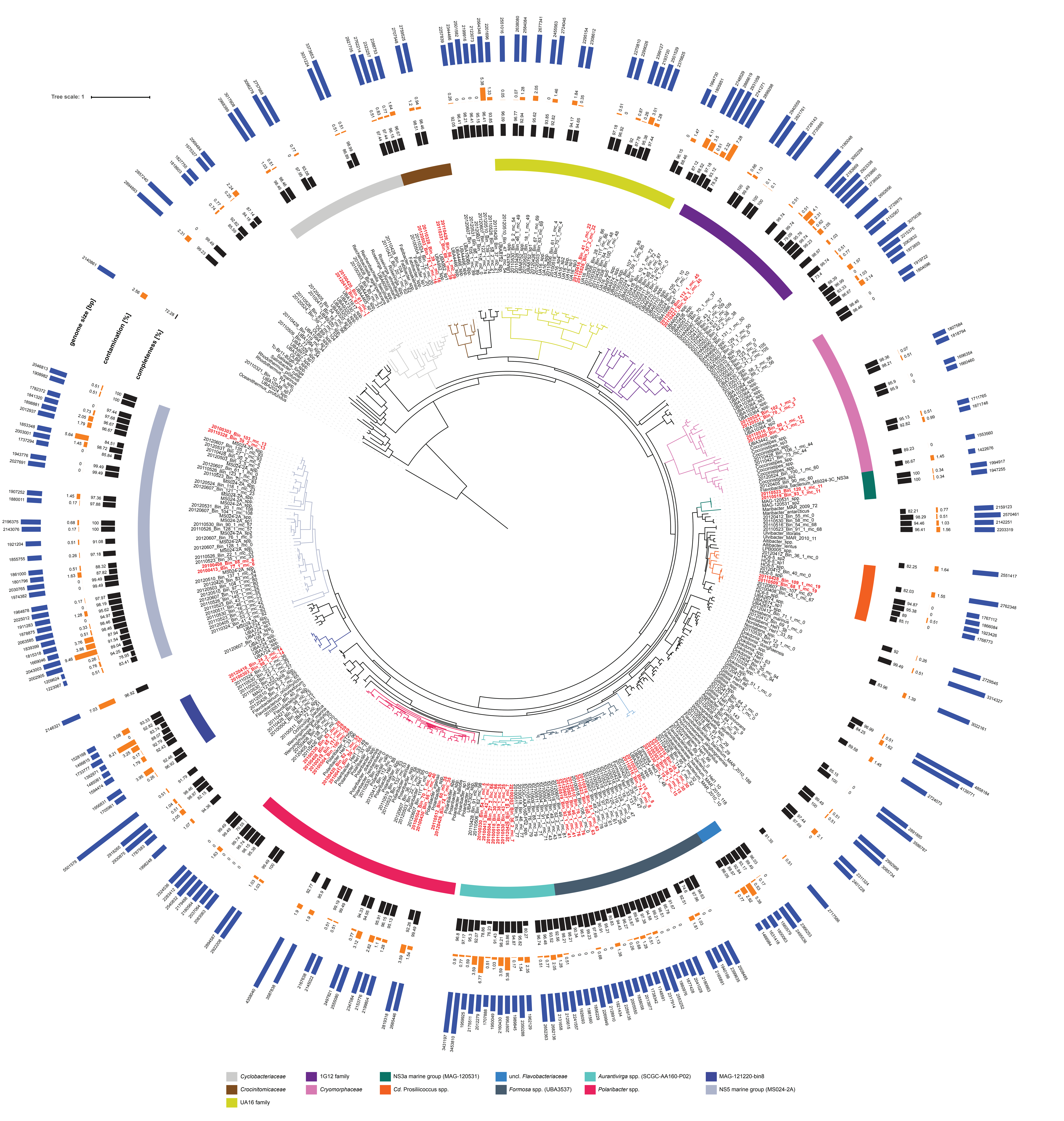

Supplementary Figure S1 Detailed maximum-likelihood tree of Mash-clusters based on concatenated marker proteins according to the GTDB-Tk genome phylogeny (48). Mash-clusters (mc) were only included in the tree that contained at least two MAGs ≥70% completeness and are represented by two MAGs each. Additional 29 singleton MAGs with ≥70% completeness and ≤10% contamination were included (labeled as mc_0). Mash-clusters reaching high abundances (>5 RPKM at one time-point or 38 RPKM at all time-points combined) during the sampling period are highlighted together with their taxonomic affiliations. Black and orange colored bars depict completeness and contamination values of respective MAGs, while blue bars indicate MAG assembly sizes. Scale bar: mean number of amino-acid substitutions per site. Outgroup: *Oceanithermus profundus*.
