# Supplementary material for: In marine Bacteroidetes the bulk of glycan degradation during algae blooms is mediated by few clades using a restricted set of genes
Source: ISME J. 2019 Jul 17;13(11):2800–16. doi: 10.1038/s41396-019-0476-y (PMC6794258; doi:10.1038/s41396-019-0476-y)
Supplement: Supplementary file 3 — Supplementary Figure S2 [file 41396_2019_476_MOESM3_ESM.pdf]

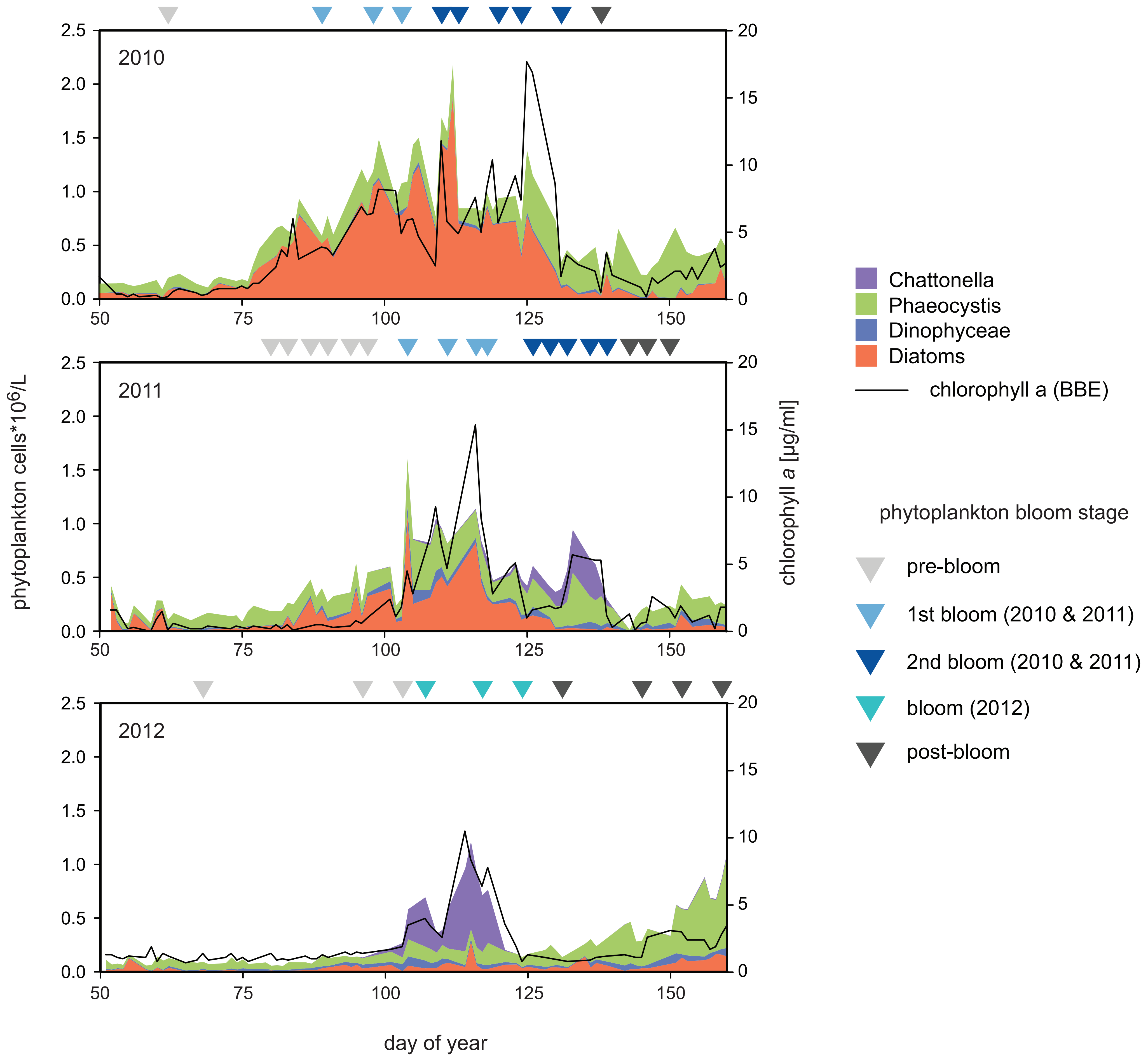

Supplementary Figure S2 Phytoplankton blooms in the years 2010 to 2012 depicted both by chlorophyll *a* and cell counts of major phytoplankton groups. Triangles on top indicate metagenome sampling time-points and are color coded based on phytoplankton bloom stage.
