# Supplementary material for: In marine Bacteroidetes the bulk of glycan degradation during algae blooms is mediated by few clades using a restricted set of genes
Source: ISME J. 2019 Jul 17;13(11):2800–16. doi: 10.1038/s41396-019-0476-y (PMC6794258; doi:10.1038/s41396-019-0476-y)
Supplement: Supplementary file 4 — Supplementary Figure S3 [file 41396_2019_476_MOESM4_ESM.pdf]

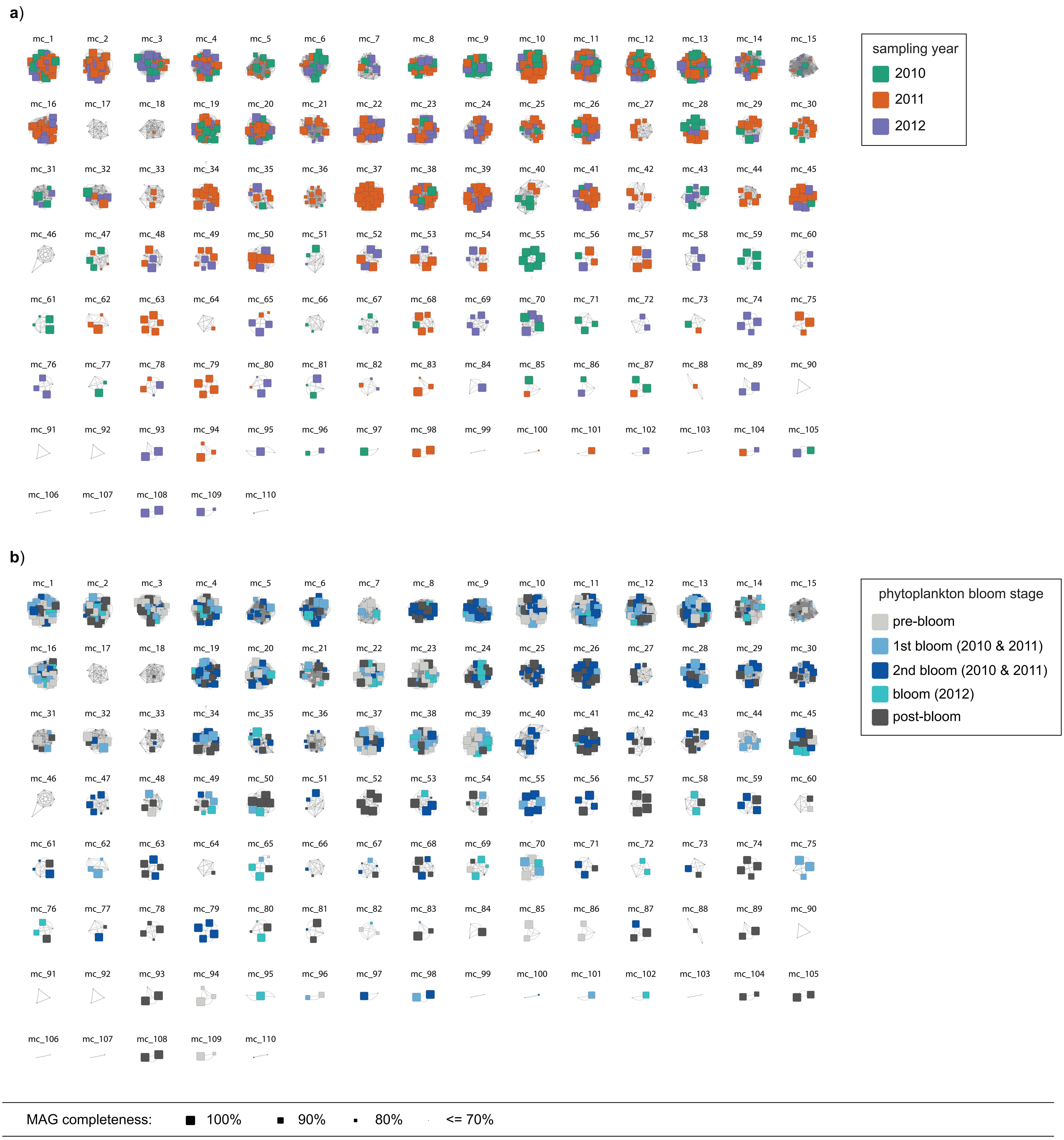

Supplementary Figure S3 Composition of all *Bacteroidetes* Mash-clusters with respect to sampling year (**a**) and phytoplankton bloom stage (**b**). Squares represent individual MAGs with sizes corresponding to completeness, while gray lines indicate Mash distances ≤0.05, and thus the approximate species connections for MAGs in each Mash-cluster. (**a**) Color coding indicates the year in which individual MAGs were retrieved. (**b**) Color coding represents phytoplankton bloom stages.
