# Supplementary material for: In marine Bacteroidetes the bulk of glycan degradation during algae blooms is mediated by few clades using a restricted set of genes
Source: ISME J. 2019 Jul 17;13(11):2800–16. doi: 10.1038/s41396-019-0476-y (PMC6794258; doi:10.1038/s41396-019-0476-y)
Supplement: Supplementary file 5 — Supplementary Figure S4 [file 41396_2019_476_MOESM5_ESM.pdf]

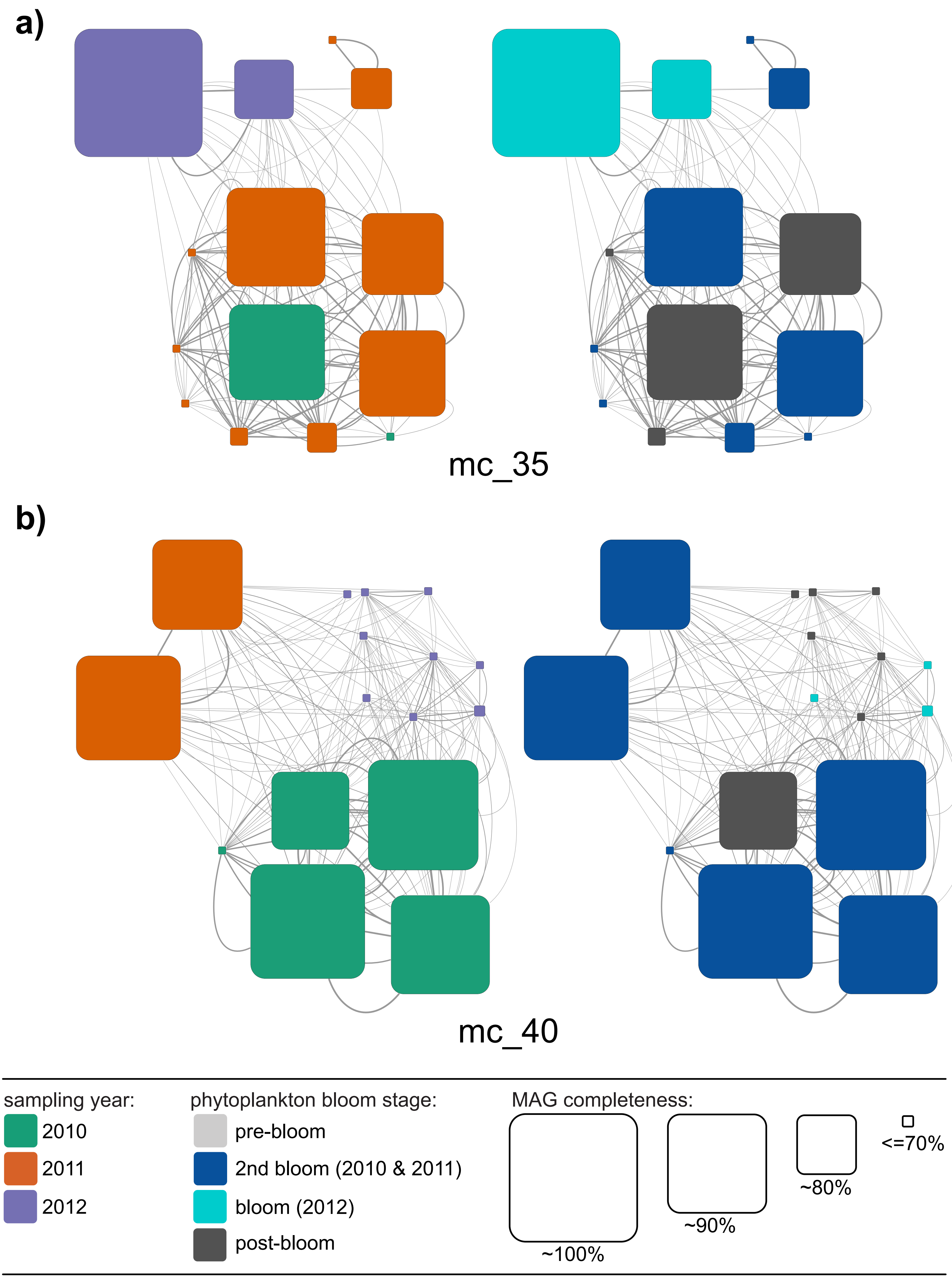

Supplementary Figure S4 *Polaribacter* Mash-clusters mc_35 **(a)** and mc_40 **(b)**. These two Mash-clusters of lesser complexity are composed of dedicated sub-clusters with respect to sampling year (left) and phytoplankton bloom stage (right).
