# Supplementary material for: In marine Bacteroidetes the bulk of glycan degradation during algae blooms is mediated by few clades using a restricted set of genes
Source: ISME J. 2019 Jul 17;13(11):2800–16. doi: 10.1038/s41396-019-0476-y (PMC6794258; doi:10.1038/s41396-019-0476-y)
Supplement: Supplementary file 6 — Supplementary Figure S5 [file 41396_2019_476_MOESM6_ESM.pdf]

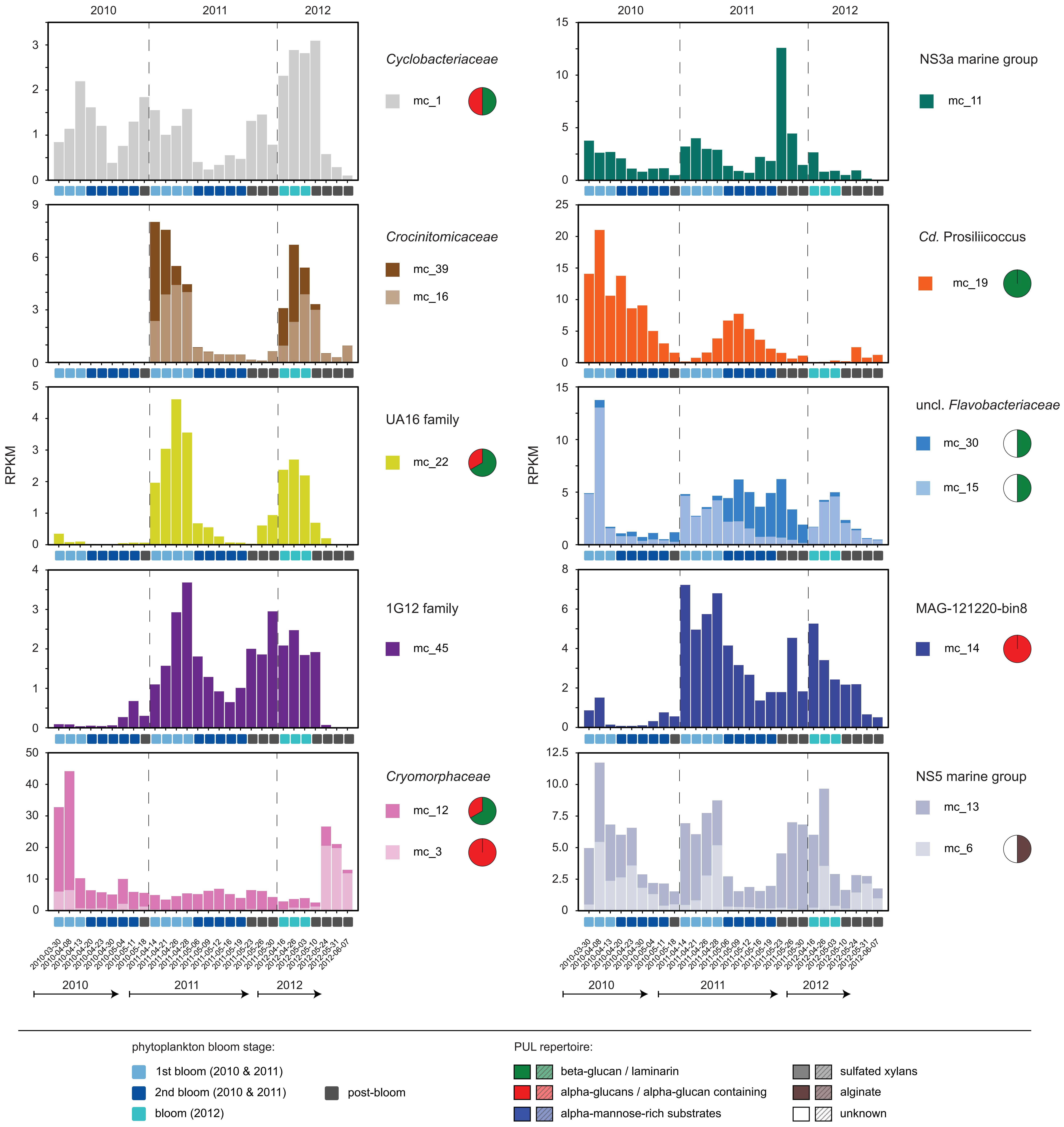

Supplementary Figure S5 Abundance pattern of Mash-clusters at bloom and post-bloom phytoplankton bloom stages. Mash-cluster abundances are shown as reads per kilobase per million (RPKM) values in all bar charts. (Mid-) bloom and post-bloom abundances are shown for all abundant Mash-clusters from clades not presented in Figure 6. Pie-charts depict predicted PUL repertoires of respective Mash-clusters.
